# Cost of rotavirus diarrhea for programmatic evaluation of vaccination in Vietnam

**DOI:** 10.1186/s12889-016-3458-2

**Published:** 2016-08-11

**Authors:** Arthorn Riewpaiboon, Sunheang Shin, Thi Phuong Mai Le, Dinh Thiem Vu, Thi Hien Anh Nguyen, Neal Alexander, Duc Anh Dang, the Rotavirus Economic Study Group, Le Huu Tho, Vu Thi Thuy, Ta Van Chan, Phu Quoc Viet, Nguyen Minh Nguyet, Le Thi Hoa

**Affiliations:** 1 Faculty of Pharmacy, Mahidol University, 447 Sri Ayutthaya Road, Ratchathevi, Bangkok 10400 Thailand; 2 MRC Tropical Epidemiology Group, London School of Hygiene & Tropical Medicine, London, UK; 3 International Vaccine Institute, Seoul, South Korea; 4 National Institute of Hygiene and Epidemiology, Hanoi, Vietnam

**Keywords:** Rotavirus burden, Acute gastroenteritis, Cost of illness, Vietnam

## Abstract

**Background:**

Rotavirus is the most common etiology of diarrhea-associated hospitalizations and clinic visits in Vietnamese children < 5 years old. To estimate the economic burden of rotavirus-associated formal healthcare encounters, an economic study was conducted.

**Methods:**

A cost-of-illness study was performed from a societal perspective. Data were collected from children below the age of five years who presented to a clinic or hospital with symptoms of acute gastroenteritis (AGE). Patient-specific information on resource use and cost was obtained through caregiver interviews and medical chart review. Costs are presented in 2014 US dollar ($).

**Results:**

A total of 557 children with symptoms of AGE were enrolled from March through June 2009, with mean age of 16.5 months. Of the 340 outpatients and 217 admitted patients enrolled, 41 % tested rotavirus positive. It was found that, from a societal perspective, the mean total cost of AGE was $175. Costs of patients with and without rotavirus were $217 and $158, respectively. From multiple regression analysis, it was found that rotavirus infection, patient age and receiving oral rehydration solution before visiting health facility had significant effect on the costs.

**Conclusions:**

This study clearly demonstrated substantial economic burden of AGE including rotavirus disease. They were significantly greater than the previously reported cost estimates in Vietnam. These updated costs of illness result in more favorable vaccine cost-effectiveness than in previous economic evaluations.

## Background

Rotavirus infection is a major reason for hospitalization and clinic visits among children under five years of age, with a significant impact on utilization of health care resources and costs, both in developed and developing countries [1–3]. Recently, two rotavirus vaccines have been licensed globally but neither has been adopted in the routine immunization program in the majority of developing countries. Decisions concerning the addition of new vaccines to national immunization program are driven by scientific, political, economic, and logistic considerations [4]. Key factors influencing such a policy decision include the economic burden of disease on health care system and society and the cost-effectiveness of a new immunization program. The cost-effectiveness of rotavirus vaccines has been evaluated more widely over the past decade [5]. In Vietnam, several groups have published the cost effectiveness of rotavirus vaccine [3, 6–8]. All the previous studies on the cost-effectiveness of rotavirus vaccine were based on the same cost estimates of rotavirus disease from the earliest study by Fischer et al. [3] which, as its authors acknowledged, had limitation in its assumptions and methodologies [3]. First, patient-specific data were obtained retrospectively by interviewing families of historical patients, in which recall bias could not be excluded. Second, per bed-day and per-visit costs were estimated based on the opinions of regional health experts rather than a properly designed institutional costing study. Third, costs were estimated for historical diarrheal patients without confirmed rotavirus etiology. Last, data were collected from the patients recruited in one province, making it problematic, as it is not representative of nationally. Aiming to update knowledge of the country’s burden of rotavirus disease, we performed a cost-of-illness study in three provinces of Vietnam, based on prospective study of rotavirus patients. In addition, we demonstrated how to use the costs in economic evaluation of vaccination program.

## Methods

### Study design

This study was designed as an incidence-based, cost-of-illness study from a societal perspective. An incidence-based approach considers only the newly occurring cases within a study period and follows them during the entire course of each episode. Costs were measured based on a micro-costing approach [9, 10]. This study was nested in a rotavirus surveillance project, which was performed in three provinces of Vietnam during 2009.

### Study population and sites

Vietnam is a developing country in Southeast Asia with a long coastline on the South China Sea, stretching more than 2400 km from a temperate climatic zone in the north to a subtropical climate in the South. The population of 89.71 million (according to the census in April 2012) includes an annual birth cohort of 1.7 million with an infant mortality rate of 15.3 per 1000 live births in 2013 [11]. The study was carried out in three provinces representing the Northern, Northeastern and Southeastern regions. Vinh Phuc in the Red River delta of Northern Vietnam, population 1.0 million; Hai Phong in the Northeastern coastal area, population ~1.9 million; and Khanh Hoa in the Southeastern coastal area, population 1.1 million. For both the rotavirus surveillance and the cost-of-illness studies, cases with symptoms of acute gastroenteritis (AGE) were enrolled among children aged less than 5 years and living in one of the five catchment areas of three provinces in Vietnam: Nha Trang city in Khanh Hoa Province, Vinh Yen city and Bin Xuyen district in Vinh Phuc province, and Kien An and Le Chan districts in Hai Phong. Catchment areas were selected considering the balance of geographic location and economic level in order to make data more representative of the whole country. It was also considered whether a population could easily be defined in terms of health care utilization pattern and was relatively stable. We invited all hospitals and major outpatient clinics that were known to serve pediatric diarrheal patients in each of the five catchment areas.

### Sample size

In order to obtain more accurate and representative cost estimates, patients were divided into groups based on the suspected cost predictors, including geographical location (province), type of patient care (inpatient, outpatient), type of facilities (private clinic, polyclinic, hospital), rotavirus in stool, patients receiving oral rehydration solution prior to institutional treatment, patients receiving breastfeeding, and child’s age. Stratified by a combination of predictor variables, at least 30 cases are needed for each patient-group [12]. Thus, we targeted at least 330 AGE episodes to be enrolled for this economic study.

### Definition

In this study, total cost of illness is defined as the sum of direct cost and indirect cost (the cost of productivity loss) starting from the onset of illness through the end of each episode. Thus, information was collected on all resource uses incurred at any health care provider/service before and after presentation to a study site, together with the resources used at the study facility. Direct costs are defined as direct medical and direct non-medical costs. Direct medical cost is the sum of treatment costs. Direct non-medical costs are for travel and meals. Indirect cost is defined as time lost by caregivers due to the child’s illness, because patients in this study are not of working age [13, 14]. Hence, caregiving time was calculated as the number of person-days that parents and/or family members spent on providing care for the sick child.

### Data and stool collection

Treatment costs prior to and after the presentation to a study site were based on the expenditure information obtained from caregiver interviews. The treatment costs at a study site (except for private clinics) were the sum of the cost of routine services at OPD/IPD, costs of medicines, and laboratory investigation costs.

Dedicated staff reviewed the address and age of each AGE patient upon presentation in order to check eligibility. Once written informed consent was obtained from parents/guardians, information was collected through caregiver interview and medical chart reviews. A pre-structured questionnaire was used in interviewing caregivers to collect demographic information and history of healthcare utilization (e.g. type of facilities where admitted or treated, and duration of stay or frequency of visit). Any out-of-pocket expenses that incurred during the course of each episode were ascertained by asking caregivers, and included treatment cost, transportation fee, and cost of meal and/or accommodation. The total time spent for caring for the sick child by all caregivers was collected to calculate time cost (or productivity loss). Trained staff reviewed medical records to extract the relevant data on medical resource uses, then recorded them on a standardized case report form. Information collected included the list and quantity of medicines, laboratory tests, and the number of days hospitalized. Family members were followed up every three days after discharge until the recovery of each episode was confirmed.

A bulk stool specimen was collected from each patient in a standardized stool container. Within 48 h of collection, stool specimens were transported to the referral location. Once a week, specimens were sent to the National Institute for Hygiene and Epidemiology (NIHE) in Hanoi and tested for rotavirus antigen by enzyme immunoassay (EIA) test using a commercial kit (DAKO, UK) under the standard operating procedure [15].

### Costing methods

Unit costs of laboratory tests and routine services were extracted from a facility-based institutional costing study performed in Khanh Hoa province [16]. The costs included material, labor and capital costs. Unit prices were adjusted by the ratio of cost to charge, resulting in unit costs for this study. These ratios were 1.18 and 1.17 for polyclinics (public clinics) and hospitals, respectively [16]. Treatment costs at a private clinic were based on the charged prices. Caregivers’ time costs were calculated according to the reference wage: 88,380 Vietnamese Dong (VND) per day [17]. The cost of rotavirus antigen testing was not included because it was a part of surveillance project (not routine practice).

Costs were estimated under the assumptions of two models: Model A classified the time loss of caregivers as indirect cost (base case) and Model B classified it as direct non-medical cost [9, 10]. All costs were originally calculated in 2009 Vietnamese Dong (VND). They were converted to 2014 values by the consumer price index of medicine and health care (2009 = 100, 2014 = 195.08) [17]. Finally, they were converted to US dollars ($) using the exchange rate of $1.00 = 21,130.5 VND [18].

### Data management and analysis

Data were double entered into a custom-made database, using Microsoft Visual FoxPro Software 9.0 platform and then exported into Microsoft Excel for verification and further analyses. IBM SPSS Statistics version 21 was used for statistical analysis [19]. Descriptive statistics were used to summarize study variables. The costs were log-transformed to meet the assumption of normal distribution. Model checks included linearity, normal distribution, multi-collinearity, influential observations, and outliers [20].

To obtain adjusted estimates of the effect of rotavirus on costs, stepwise multiple regression analysis was employed [20]. Independent variables with probability values of *F* statistics ≤ 0.05 were entered. Sensitivity analysis was conducted to measure the effect of cost classification; Models A and B, classifying costs of caregiver time (informal care) as indirect and direct, respectively. In addition, the costs were measured based on different economic evaluation methods.

## Results

From March till June 2009, 557 episodes were included in the analysis. Characteristics of patients are presented in Table 1. Forty-one percent of AGE patients had rotavirus in stool (positive EIA test) and the average patient age was 16.5 months. Comparing patients with and without rotavirus in stool, there was no statistically significant difference in terms of age, gender, or receiving primarily breast milk. However, there were differences in the proportion rotavirus positive in terms of prior receipt of whether oral rehydration solution had been given prior to presentation (53.0 % rotavirus positive) or not (35.9 %). Similarly, inpatients were more likely to be rotavirus-positive (55.3 %) than outpatients (31.8 %). Resource utilization and costs are presented in Table 2. Caregiving days of parents or relatives of patients with rotavirus were statistically significantly higher than those of patients without rotavirus. Again, direct medical cost, direct cost, indirect cost and total cost of illness of patients with rotavirus were statistically higher than those of those without rotavirus. Only direct non-medical cost (not including cost of caregiver time) was not statistically significantly different. Costs of patients with rotavirus-positive were higher than those of rotavirus-negative in all age groups. Older patients had lower costs than younger patients, whether rotavirus-positive or-negative (Table 3). To confirm the effect of rotavirus on costs, multiple regression analysis was conducted. Rotavirus status and other potential predictors of direct medical cost, direct cost and total cost of illness are in Table 4. Fitted models are presented in Table 5. The models include rotavirus in stool, age and receiving ORS. Power of explanation (adjusted *R*^2^) was in range of 7–13 %.

**Table 1:** Characteristics of patients

		Rotavirus in stool		Total
		No	Yes	
Age (month)	Mean	16.67	15.63	16.51
	Median	12.00	13.00	13.00
Gender; female	Female	137	95	232
		59.1 %	40.9 %	100.0 %
	Male	192	133	325
		59.1 %	40.9 %	100 %
Child primarily breastfed; yes	Yes	306	213	519
		59.0 %	41.0 %	100.0 %
	No	21	14	35
		60.0 %	40.0 %	100 %
Receiving oral rehydration solution before visiting the study facility^a^; yes	Yes	78	88	166
		47.0 %	53.0 %	100.0 %
	No	250	140	390
		64.1 %	35.9 %	100 %
Health facility type^a^	Private clinic	33	16	49
		67.3 %	32.7 %	100.0 %
	Polyclinic	97	53	150
		64.7 %	35.3 %	100.0 %
	Hospital outpatient	102	39	141
		72.3 %	27.7 %	100.0 %
	Hospital inpatient	97	120	217
		44.7 %	55.3 %	100.0 %
Type of patient^a^	Outpatient	232	108	340
		68.2 %	31.8 %	100.0 %
	Inpatient	97	120	217
		44.7 %	55.3 %	100.0 %
Province^a^	Hai phong	106	69	175
		60.6 %	39.4 %	100.0 %
	Vinh phuc	140	55	195
		71.8 %	28.2 %	100.0 %
	Nha trang	83	104	187
		44.4 %	55.6 %	100.0 %
	Total	329	228	557
		59.1 %	40.9 %	100.0 %

^a^Significant difference; Chi-Square Tests at *p* < 0.05

**Table 2:** Resource utilization and costs (2014 USD)

	Rotavirus in stool								
	No (*n* = 329, 59.1 %)			Yes (*n* = 228, 40.9 %)			Total (*n* = 557)		
	Mean	Median	SE	Mean	Median	SE	Mean	Median	SE
Length of stay of inpatients (days)	5.81	5.00	0.31	5.55	5.00	0.23	5.68	5.00	0.18
Caregiving (person-day)^a^	7.44	6.00	0.28	9.44	8.00	0.36	8.06	7.00	0.21
Total direct cost (not including informal care)^a,b^	96.99	42.69	6.67	139.80	129.97	8.14	108.92	49.96	4.84
Total direct medical cost^a,b^	75.12	16.37	6.40	117.71	104.55	7.92	87.13	16.74	4.68
Direct nonmedical cost (not including informal care)	21.87	18.47	1.15	22.09	18.75	1.27	21.79	18.47	0.82
Indirect cost (with informal care)^a,b^	60.73	48.95	2.32	77.03	65.27	2.97	65.74	57.11	1.73
Total cost of illness^a,b^	157.72	97.97	8.17	216.83	196.44	10.37	174.66	107.38	6.06

^a^Statistically significant difference; Mann-Whitney U Test at *P* < 0.05

^b^Statistically significant difference; T-Test of natural log form at *P* < 0.05

**Table 3:** Average cost of illness (2014 USD) classified by age, rotavirus infection and cost component

Age^a^ (years)	Rotavirus in stool	Number of cases	Total direct medical cost	Total direct cost	Total cost of illness
1	No	167	87.74	115.45	181.46
	Yes	106	125.11	152.17	231.64
2	No	94	69.78	86.73	147.53
	Yes	87	124.75	143.13	222.19
3	No	33	70.78	86.41	140.80
	Yes	19	81.64	100.95	172.67
5	No	35	33.34	46.43	87.81
	Yes	16	73.38	85.94	142.04

^a^no patient aged 4 years

**Table 4:** Potential predictor variables for multiple regression analysis

Dependent variables	
LnDMC	Natural log of direct medical cost
LnDC	Natural log of direct cost (not include informal care)
LnTC	Natural log of cost of illness
Potential predictor variables	
Receiving oral rehydration solution before visiting the study facility	Receiving = 1 (30 %), no = 0 (70 %)
Rotavirus in stool	Positive = 1 (41 %), negative =0 (59 %)
Child age (months)	Mean age in months (16.27)
Child receiving primarily breastfed	Receiving = 1 (94 %), no = 0 (6 %)

Note: Type of patients and health facility were tested in the model separately

**Table 5:** Regression models investigating effect of rotavirus infection on the costs

Predictor variable	Unstandardized coefficients		
	Direct medical cost	Direct cost	Total cost of illness
Constant	3.437	4.318	4.950
Rotavirus in stool	.591	.446	.320
Child age (months)	-.020	-.028	-.017
Receiving oral rehydration solution before visiting the study facility	.299	n/s	.212
Adjusted R^2^	.068	0.095	0.129

n/s = not statistically significant

Table 6 shows the sensitivity of the results to model choice. Model A is the base case, with informal care costs classified as indirect, rather than direct non-medical (Model B). Both models were used to estimate cost-effectiveness in terms of a) case avoided or life saved, and also b) years of life gained. In societal-perspective cost-utility analysis (CUA), only direct costs are included [21]. Direct costs are necessarily higher when they are defined more widely to include informal care (Model B) and, in turn, this increases the incremental cost-effective ratio (ICER), of which cost is the numerator. Hence, differences in classifying informal costs care have affected the economic evaluation results.

**Table 6:** Cost of rotavirus diarrhea classified by economic evaluation methods from societal perspective (2014 USD)

	Cost by evaluation methods		
	CEA1^a^	CEA2^b^	CUA^c^
Model A: Cost of informal care as a part of indirect cost.			
1. Direct cost	139.8	139.8	139.8
1.1 Direct medical cost	117.71	117.71	117.71
1.2 Direct nonmedical cost	22.09	22.09	22.09
2. Indirect cost	77.03	n/a	n/a
Model B: Cost of informal care as a part of direct nonmedical cost.			
1. Direct cost	216.83	216.83	216.83
1.1 Direct medical cost	117.71	117.71	117.71
1.2 Direct nonmedical cost	99.12	99.12	99.12
2. Indirect cost	0	n/a	n/a

Notes: ^a^CEA1 = cost-effectiveness analysis in terms of case avoided or life saved

^b^CEA2 = cost-effectiveness analysis in terms of life years gained

^c^CUA = cost-utility analysis in terms of quality-adjusted life year (QALY) or disability-adjusted life

Year (DALY)

n/a = not applicable; all costs were estimated based on societal perspective

## Discussion

Focusing on the cost of rotavirus diarrhea in Southeast Asia, studies in Indonesia, Malaysia and Vietnam have been published in international journals [3, 22–24]. In a study conducted in Indonesia in 2007 [22], parents of 1349 patients were interviewed during outpatient visits, while inpatients were interviewed on discharge and again two weeks later. The study covered direct medical costs, direct non-medical costs and indirect costs (time taking care of sick children). Direct medical costs were based on actual payments made by the patients. In Malaysia, Lee et al. (2002) measured the cost of inpatients from a hospital perspective. Three hundred and ninety-three patients were included in the study. The costs included were treatment costs or direct medical costs, covering only patient service departments. Costs of supporting departments (e.g. administration) were not included [23]. Another study conducted in Malaysia during August 2006 and July 2007 measured out-of-pocket costs [24]. The study covered 260 patients and included expenses for medical consultations, medication prior to hospitalization, hospital bills, any special food or diet, transportation, extra diapers, productivity loss for caregivers, and other costs incurred by family members. In Vietnam, a 2003 study enrolled 90 patients [3] and included direct medical costs, direct non-medical costs, and indirect costs (time taking care of sick children). Direct medical costs were estimated per day and per visit, by interviewing government officials. For direct non-medical costs and indirect costs, parents were interviewed.

Due to the different study designs and data limitations, it was not feasible to directly compare costs between studies. For direct medical or treatment costs, none of the previous studies used direct measurement of the actual costs over the whole hospital (service and supporting departments). This could be a limitation in terms of economic evaluation from a societal perspective. Therefore, our study covered both actual and complete cost of the hospitals with considerable sample size.

In terms of cost drivers, in addition to rotavirus infection, it is interesting to notice a similarity between the study in Canada and our one, namely that age was significant predictor variable [25]. The history of receiving ORS was an additional significant predictor variable in our analysis (Table 5). In these models, coefficients of determination indicating power of explanation were small (0.068–0.129). In the exploration, we fitted a model by adding health facility types (private clinic, polyclinic and hospital) and found an increase of the coefficient to 0.885. However, with significant effect of the facilities, rotavirus infection did not show the effect. Our interpretation is that rotavirus infection has effects on the cost but much less than the types of services at different levels of health facilities. In real-life practice, rotavirus infection is not investigated by laboratory test: laboratory investigations reported here were done as part of the research project. Therefore, in day-to-day practice, physicians provide treatment based on clinical symptoms, patient and health system factors.

In terms of cost driver analysis, we used stepwise multiple regression analysis. We had limited ability to validate the model using out-of sample data. Costs are a major concern in economic evaluation. Reduction in cost of illness increases net cost and raises the cost-effectiveness ratio, making the intervention less attractive economically [26]. As shown in Table 6, we found a substantially larger cost burden of rotavirus disease in Vietnam than Fischer et al. in 2004 [3]. The 2014 values of the 2004 study were USD88, USD18, USD16 and USD21 for in-patients, outpatients at hospitals, polyclinics and private clinics, respectively. Several factors might have contributed to this. First, costs were estimated based on the data collected from patients with rotavirus infection in a prospective manner, whereas the earlier study was based on historical patients. Second, we followed up each patient during the entire period of an episode starting from the onset of disease until the recovery. Nearly one half of the patients enrolled in our study reported that they had sought health care prior to presentation to a study health facility. Relevant costs associated with any health care utilization prior to or after presentation to a study health facility were traced, identified, and included in our cost estimation. Such an intensive follow-up could not have been made in the previous study since data were obtained from historical episodes. Third, we calculated unit costs per clinic visit and per bed day based on an actual study from Vietnam [16], whereas the previous study was based on the opinions of local health officials. Last, our study was based on the data from multiple study sites throughout three provinces in different regions of Vietnam whereas the earlier cost estimates were based on the data from one province [3]. However, a limitation of our study was the absence of patients with mild symptoms who self-treated and did not come to the study sites. This limits the generalizability in terms of economic burden. Information on the economic burden of an illness is important in setting priorities, financial and budgeting management, and efficiency management [10]. Cost of illness is a measurement of an illness’ economic burden on society. Hurdles in conducting cost-of-illness studies include the absence of reliable local data, and conceptual or methodological issues in costing [27]. The latter include controversies in including productivity cost [28], costing methods (e.g., human capital approach versus friction cost method [29]), and reference values (e.g. discount rate [30]). Whether to classify caregivers’ time as direct non-medical or indirect cost has also been controversial [10]. The point at issue is double counting because time cost can be included as either monetary value or health state (e.g., quality adjusted life year) [9]. In a review of cost-utility analysis published during 1976 and 1997, 5.7 % of the papers included caregiver costs [31]. In a similar economic evaluation, a study in Thailand classified caregiver costs as direct-nonmedical [32], while another in Indonesia classified them as indirect costs [22]. One study omitted mortality costs, to avoid double counting, but did not mention the costs of caregiver time loss [33]. Another included them as indirect costs without identifying their components [34]. Hodgson concluded that cost-utility analysis from a societal perspective should exclude indirect costs of morbidity and mortality (but not that of caregivers) [26]. In summary, previous studies varied in terms of inclusion and classification of indirect costs in their cost-utility analysis. This could affect their results in terms of the incremental cost-effectiveness ratio (ICER). If the ICER is less than the threshold of willingness-to-pay (for instance, 1 per capita gross domestic product) for the health intervention evaluated, the intervention is accepted, and conversely. For instance, in a study on economic evaluation of varicella vaccination, the vaccine was not acceptable for a base-case analysis (not including the indirect cost). However, when the indirect cost was included, the vaccine is acceptable [35].

WHO guideline recommends that time costs should be reported separately from other cost estimates in cost of illness studies. The guideline also recommends that cost-effectiveness or cost-utility analysis should be conducted both with and without these time costs [14]. Thus, in our analysis, the cost of rotavirus disease was based on the two different models of cost classification that caregiver’s time was included as indirect cost versus non-medical direct costs (Table 6).

## Conclusion

This study clearly demonstrated a substantial cost burden of rotavirus disease to society in Vietnam. This burden was significantly greater than found previously [3], implying a more favorable cost effectiveness outcome of rotavirus vaccination in the country. As the first step, for Vietnam government’s policy decision on whether to implement rotavirus vaccination, an economic evaluation of the vaccine should be repeated in the Vietnamese context based on this updated cost information of rotavirus disease.

## Abbreviation

AGE, acute gastroenteritis; CEA, cost-effectiveness analysis; CUA, cost-utility analysis; DALY, disability-adjusted life year; EIA, enzyme immunoassay; NIHE, National institute for hygiene and epidemiology; ORS, oral rehydration salt; QALY, quality-adjusted life year; VND, Vietnamese Dong
